# Home-Based Pediatric Palliative Care and Electronic Health: Systematic Mixed Methods Review

**DOI:** 10.2196/16248

**Published:** 2020-02-28

**Authors:** Heidi Holmen, Kirsti Riiser, Anette Winger

**Affiliations:** 1 Oslo Metropolitan University Oslo Norway

**Keywords:** eHealth, home-based, pediatric palliative care, pediatric, children, family, communication, palliative care

## Abstract

**Background:**

Children and families in pediatric palliative care depend on close contact with health care personnel, and electronic health (eHealth) is suggested to support care at home by facilitating their remote interactions.

**Objective:**

This study aimed to identify and review the use of eHealth to communicate and support home-based pediatric palliative care and appraise the methodological quality of the published research.

**Methods:**

We conducted a convergent, systematic mixed methods review and searched Medical Literature Analysis and Retrieval System Online (Medline), EMBASE, PsycINFO, Cochrane Library, Cumulative Index to Nursing and Allied Health Literature (CINAHL), Web of Science, and Scopus for eligible papers. Studies evaluating 2-way communication technology for palliative care for children aged ≤18 years and applying quantitative, qualitative, or mixed methods from 2012 to 2018 were eligible for inclusion. Quantitative and qualitative studies were equally valued during the search, screening, extraction, and analysis. Quantitative data were transformed into qualitative data and analyzed using a thematic analysis. Overall, 2 independent researchers methodologically appraised all included studies.

**Results:**

We identified 1277 citations. Only 7 papers were eligible for review. Evaluating eHealth interventions in pediatric palliative care poses specific methodological and ethical challenges. eHealth to facilitate remote pediatric palliative care was acknowledged both as an intrusion and as a support at home. Reluctance toward eHealth was mainly identified among professionals.

**Conclusions:**

The strengths of the conclusions are limited by the studies’ methodological challenges. Despite the limitless possibilities held by new technologies, research on eHealth in home-based pediatric palliative care is scarce. The affected children and families appeared to hold positive attitudes toward eHealth, although their views were less apparent compared with those of the professionals.

**Trial Registration:**

PROSPERO CRD42018119051; https://tinyurl.com/rtsw5ky

## Introduction

### Background

Pediatric palliative care is a heterogeneous field concerning children with various life-threatening or life-shortening conditions from birth until young adulthood [1]. Pediatric palliative care is provided regardless of diagnosis, and the aim is increased quality of life; “Palliative care for children is the active total care of the child’s body, mind and spirit, and also involves giving support to the family” [2]. There is no definite number of children in need of pediatric palliative care. Global estimates suggest that between 113 per 10,000 children in Zimbabwe and 20 per 10,000 children in the United Kingdom are in need of specialized or generalized palliative care [3]. The complexity of the needs of these children and their families makes them dependent on multidisciplinary efforts to manage symptoms and provide psychosocial care and support [1,4]. These children often experience pain related to medical treatment that is frequently worsened in a hospital—a nonfamiliar environment [5]. Previous studies support pediatric palliative care at home, where children and their families are most at ease [4,6], to improve their quality of life [4] and their quality of care [7,8]. Home-based pediatric palliative care should involve a specialized interdisciplinary team that is often organized in specialized care [4,9]. To meet the care needs of these children and their families, the professionals involved must directly communicate not only with the child and his or her family but also with one another [10]. However, communication is suggested to be a core challenge in pediatric palliative care [7].

Electronic health (eHealth) systems facilitate remote communication to provide care at home without requiring that patients or health care personnel (HCP) travel. eHealth is defined as “the use of information and communication technology for health” [11]. The relevance of eHealth in home-based pediatric palliative care has been highlighted, thus suggesting that eHealth can improve patients’ quality of care [7]; however, further research is warranted as the full potential of health technology has not yet been realized [6]. A recent case report suggested how mobile technology provides a platform for affordable and high-quality communication such as through videoconferences [12]. The factors in favor of eHealth in home-based pediatric palliative care are ease of use, patient and clinician satisfaction, and the potential for saving travel time and money for patients and HCP [6,12]. Age-based preferences regarding technology and communication with clinicians should guide the development of new technology [7], particularly relevant in pediatric palliative care [7].

Bradford et al [6] conducted a systematic review in 2013 to summarize the evidence for home-based telehealth in pediatric palliative care. They emphasized the logistical and ethical issues regarding research that involves this vulnerable group by highlighting the importance of research that minimizes patients’ burdens [6]. This emphasis is supported by the conclusions of a general review regarding research on pediatric palliative care [13]. Despite the rapid development of technology in general, the current field of research on eHealth in home-based pediatric palliative care is rather scarce and lags behind. To ensure that home-based pediatric palliative care supported by eHealth is evidence based, we argue that an updated review of evidence published after Bradford et al’s review in 2013 was necessary [6].

### Aim

This study aimed to identify and review the use of eHealth to communicate and support home-based pediatric palliative care and appraise the methodological quality of the published research.

## Methods

### Design

This systematic mixed methods review is based on a convergent design [14]. Following the Preferred Reporting Items for Systematic Reviews and Meta-Analyses guidelines [15], we applied systematic database searches, and we integrated studies regardless of their design, and qualitative and quantitative methods were equally valued.

### Protocol and Registration

The scope and aim were developed and discussed within our research group before a protocol was written. The rationale for conducting a mixed methods approach was based on the limited existing evidence on home-based pediatric palliative eHealth, and thus, a single method review would not sufficiently clarify the evidence within the field. The review protocol was published in the International Prospective Register of Systematic Reviews (PROSPERO), ID: CRD42018119051 [16].

### Information Sources, Search, and Eligibility Criteria

To ensure a comprehensive search, we used the sample, phenomenon of interest, design, evaluation, and research type (SPIDER) search tool to identify targets and search terms [17]. Furthermore, 2 research librarians assisted in the development of a search string and tailored each string to individual databases. The systematic search was prepared in November 2017—followed by an updated search in December 2018—in the following databases: Medical Literature Analysis and Retrieval System Online (search string in Multimedia Appendix 1), EMBASE, PsycINFO, Cochrane Library, Cumulative Index to Nursing and Allied Health Literature, Web of Science, and Scopus.

Specific inclusion and exclusion criteria were applied according to the SPIDER framework (Table 1). As eHealth may nevertheless be considered a relatively new field of research, we anticipated smaller studies using noncontrolled designs; therefore, studies were included regardless of their design. We included papers published after February 22, 2012, as our review builds on a previous review [6]. There were no language restrictions, and we exclusively included papers published in peer-reviewed journals. We excluded both letters and editorials.

### Study Selection

All citations were assessed independently by 2 researchers according to the inclusion and exclusion criteria set a priori (HH assessed all citations, whereas AW and KR split the citations and each assessed one half). Disagreements were resolved by discussion among the 3 researchers, and no disagreements required an independent researcher. All citations were screened through their titles and abstracts to exclude those that clearly did not meet our inclusion criteria before reading the remaining papers in full to assess their relevance to our aim.

**Table 1:** Sample, phenomenon of interest, design, evaluation, and research type framework used to identify targets.

Framework criteria	Target
Sample	Children (aged 0-18 years) with palliative care needs, their families, and involved health care personnel
Phenomenon of interest	Home-based eHealth^a^ systems as facilitators of improved care and communication, with any 2-way eHealth communication component as the major intervention of interest
Design	Pilot and feasibility studies, field studies, case studies, cross-sectional studies, cohort studies, case-control studies, randomized controlled trials, observational studies, and all studies using a qualitative design
Evaluation	Descriptive evaluations with experiences, perceptions, and effects; any health-related outcomes (both self-reported and objective measures); and those evaluating the eHealth component were included
Research type	Qualitative, quantitative, or mixed methods designs

^a^eHealth: electronic health.

### Data Extraction

The results of the eligible papers were extracted using the following structured form based on the SPIDER framework [17]: author, country, sample, phenomenon of interest, research type and design, evaluation, and results.

### Methodological Appraisal

To appraise the methodological quality, we used the standardized checklists according to the designs of our primary studies, which are available from Joanna Briggs Institute [18]. All studies were independently assessed by 2 reviewers (HH and KR), whereas a third researcher (AW) assisted with any disagreements that arose.

### Analysis and Qualitative Synthesis

The studies were summarized descriptively and in tables. As a first step in the analytical process, all included papers were read in full by the 3 researchers, and the results sections of all papers were extracted as data for our analyses, regardless of their initial design. We transformed all quantitative data into qualitative data [14] and applied line-by-line coding, inspired by the thematic synthesis described by Thomas and Harden [19]. A thematic synthesis has been used in line with the convergent design of mixed methods reviews [20]. The 3 researchers individually coded all the material before discussing the codes and agreeing upon descriptive categories and conceptual themes. The heterogeneity of the designs, interventions, and methodological quality did not allow for any pooled statistics or meta-analyses.

## Results

### Characteristics of the Included Studies

**Figure 1:**
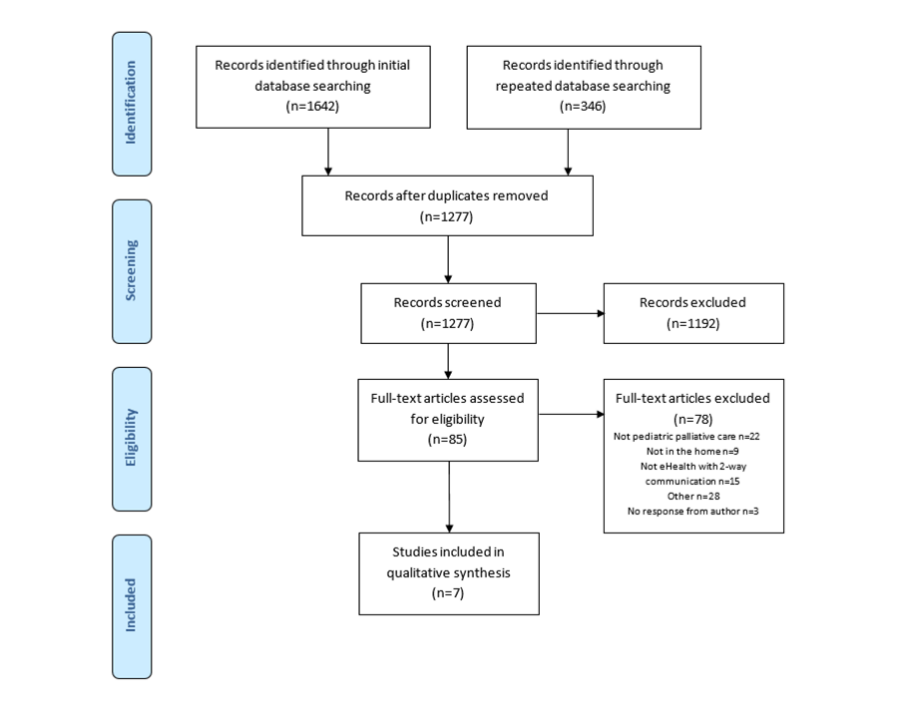
Flow chart of the systematic search process.

The initial search identified 1642 citations, and the repeated search identified 346 new citations (Figure 1). After duplicates were removed, 1277 titles and abstracts were screened according to the inclusion criteria. The remaining 85 papers were read in full. We contacted 5 authors to clarify the study details and sent 1 reminder to those who did not reply. Two authors replied positively to our requests. We included 7 papers [21-27] in our review (Tables 2 and 3).

**Table 2:** Study sample and phenomenon of interest.

Study	Country	Sample	Phenomenon of interest
Bradford et al [21]	Australia	14 caregivers (11 mothers and 3 fathers) to children aged 0 to 18 years (10 girls and 4 boys) diagnosed with a life-limiting condition (neurological, oncological, metabolic, genetic, and cardiac)	The Home Telehealth Program from the family perspective^a^
Bradford et al [24]	Australia	10 palliative care professionals (medical, nursing, and allied health) in a tertiary pediatric hospital	The Home Telehealth Program from the health care perspective^a^
Bradford et al [23]	Australia	95 home video consultations over a 2-year period	The Home Telehealth Program from an economic perspective^a^
Bradford et al [22]	Australia	100 consultations (50 telemedicine consultations during home visits and 50 face-to-face consultations); 33 patients in telehealth and 48 face-to-face consultations	The Home Telehealth Program from a consultation process perspective^a^
Harris et al [25]	England	32 families of children with life-limiting illnesses (severe cerebral palsy, intractable epilepsy, and metabolic and genetic disorders); both parents contributed in 4 families, only fathers in 4 families, and only mothers in 24 families	MyQuality online tool allows families to identify, describe, prioritize, and monitor the issues that most strongly affect their quality of life and share this information with their HCP^b^ and other professionals
Katalinic et al [26]	Australia	14 patients (gender not given) with life-limiting illnesses, with a mean age of 6 years (SD 6), and 6 professionals (staff specialist, occupational therapist, social worker, and clinical nurse specialist)	iPad for videoconferences between families and HCP; iPad had individualized content (apps) not described in the paper
Levy [27]	Scotland	14 professionals caring for children with palliative and complex care needs (7 pediatric outreach oncology nurses, 4 medical consultants, 2 specialist nurses, and 1 outreach worker)	Care at home pilot from the HCP perspective; videoconferences through laptop computers with external webcams and headsets for increased consultation quality

^a^The Home Telehealth Program consisted of videoconferences to provide specialist consultations in families’ homes, with a focus on symptom management, clinical changes, consequences for care plans, and emotional support for caregivers [21].

^b^HCP: health care personnel.

The studies applied various designs (Table 3); 1 study aimed for a controlled design [21] but was forced to end recruitment prematurely because of unanticipated patient deaths. The participants were mainly HCP who discussed technology on behalf of children and their families in pediatric palliative care. All studies evaluated an intervention. To grasp the intervention evaluated by Bradford et al [21-24], data were extracted from the primary publication [28]. Likewise, for the paper by Levy [27], the intervention was described elsewhere [29]. Overall, 6 of the 7 papers reported on videoconferences as the primary method for remote communication [21-24,26,27], whereas 1 study evaluated a Web-based tool [25]. One research group in Australia conducted 4 of the included studies [21-24], whereas the 3 remaining studies originated from 3 separate research groups: 1 also in Australia [26] and 2 in the United Kingdom [25,27].

### Methodological Appraisal

The methodological appraisal revealed several shortcomings (Multimedia Appendix 2). A total of 3 studies [21,25,26] were assessed using the checklist for quasi-experimental designs [30]. The lack of control groups and clarity regarding any comparison increased the likelihood of bias. Furthermore, 2 studies [24,27] were appraised using the qualitative research checklist [31]. We did not find a philosophical perspective in either, although the chosen methodology seemed appropriate. Neither study addressed the researcher’s influence on the research, but both adequately presented the participant’s voices. One study [23] was evaluated through the economic evaluation checklist [32] where all items were satisfactorily covered except for the item covering whether or not the intervention’s clinical effectiveness was established—in this case, videoconferencing in home-based pediatric palliative care. Finally, 1 study [22] was appraised using the checklist for case-control studies [33], where most items were satisfactorily covered. None of the included studies were excluded based on the methodological appraisal.

### Qualitative Synthesis

The qualitative synthesis resulted in the following 3 themes: *eHealth at home*, *technological features*, and *system for eHealth* (Table 4).

**Table 3:** Study design, evaluation, and results.

Study	Design	Evaluation	Results
Bradford et al [21]	Non-randomized pilot study	Follow–up after 10 weeks and primary outcome was QOLLTI-F^a^	No differences in QOLLTI-F scores between caregivers in control and intervention groups
Bradford et al [24]	Qualitative interview study	Grounded theory analysis of semistructured interviews	4 themes: managing relationships (specialist teams valued more than community-based teams), expectations from clinicians (high expectations vs low uptake), coordination (service and support), and telehealth compromise (telehealth was inferior to face-to–face consultations)
Bradford et al [23]	Cost-minimization analysis	The costs of the Home Telehealth Program compared to potential in-person consultations costs, based on clinician’s time and travel.	Air travel (n=24) significantly affected the costs. The mean intervention cost was Aus $ 294 and required no travel. Mean cost per outpatient consultation was Aus $ 748. The mean cost per home consultation was Aus $ 1214.
Bradford et al [22]	Case-control study	A 14-item checklist of a pediatric palliative care consultation was constructed. Each item scored 1 point if it was documented.	The median quality score for the face-to-face consultations was 7; the median score for the telemedicine consultations was 6. There was no significant difference between the quality scores in the 2 groups.
Harris et al [25]	Longitudinal, multisite mixed methods study	Follow-up 12 weeks. Evaluated patterns of website use (parameters, frequency, and duration), FES^b^, and semistructured interviews with family users.	23 out of 32 families used MyQuality with a mean of 106 days (min-max 2-301), including 2 or 3 parameters (min-max 1-15), most commonly seizures, constipation, pain, and sleep problems. Mean FES scores increased over time. Interview feedback confirmed the website’s acceptability and ease of use.
Katalinic et al [26]	Case study	Follow-up 12 weeks; staff and patients (by proxy) evaluated usability, usefulness, and clinical advantages of using the iPad	iPad’s primary uses: videoconferences for clinical review, case conferences, and bereavement follow-up; iPad’s secondary uses: email, internet search, socialization apps, relaxation and mood apps, and children’s movies and electronic books
Levy [27]	Qualitative interview study	Data were analyzed for common themes	Significant differences between the way telehealth was explored and used within the public and voluntary sectors. Clear benefits in and potential risks of telehealth to both patients and own practice.

^a^QOLLTI-F: Quality of Life in Life-Threatening Illness–Family.

^b^FES: Family Empowerment Scale.

**Table 4:** Themes and categories developed in the thematic qualitative synthesis.

Theme	Category
eHealth^a^ at home	Support for the child The parent perspective Support for the family
Technological features	Usability Means to communicate Technology as a care facilitator
System for eHealth	System resources HCP^b^ as part of the system

^a^eHealth: electronic health.

^b^HCP: health care personnel.

#### Electronic Health at Home

This theme concerns the experiences related to families who use eHealth technology for support at home, for which we developed the following 3 categories: support for the child, the parent perspective, and support for the family.

#### Support for the Child

Pediatric palliative care includes children in the age range of 0 to approximately 18 years. As children age, they may become more autonomous users of technology depending on their physical disability or mental capacity. Older children handled the technology more easily than their parents, and they valued the access they had to HCP without their parents being present [27]. Other quotes from HCP indicated greater reluctance toward eHealth, whereas an illness itself was identified as a potential intrusion that threatened a child’s autonomy and independence [24].

Regardless of technology, these children need an individual approach. Both the child’s and family’s preferences as well as the former’s care needs can guide the tailoring of eHealth technology to facilitate individual perspective, especially the therapeutic relation [24]. This relation seemed to be strengthened by the ability to see one another, and some HCP preferred eHealth systems with videoconferences over telephone calls [24,27]. However, there were examples of how HCP tended to overuse eHealth and provide children and families with technology rather than focus on whether or not they actually needed it. HCP expressed that unclarified expectations might explain some of the overload [24].

#### The Parent Perspective

HCP referred to how they perceived the parents’ acceptance of the technology [21,24] and reported that managing technological communication devices in addition to the burden of having a child with palliative care needs seemed demanding of the parents. Parents’ self-reported physical and emotional health were generally negative, and their quality of life did not seem to change over time among those who were given access to eHealth support or among the control group [21]. In the same study, parents found that the quality of care, satisfaction with care, and environment for care were equally positive regardless of the presence of eHealth technology.

One study found that parents generally considered the technological systems easy to set up. Moreover, they valued that the observations of the child were systematically recorded and visualized [25]. These observations increased both parents’ and HCP’s understanding of each child’s status and may have led to changes in care plans [25].

Some parents were not comfortable being on video, which was a barrier to the use of the eHealth communication systems that rely on videoconferencing [24]. Accordingly, HCP reported that the consultations became distressing for parents, which made interactions with HCP less fruitful. This was particularly evident when sensitive topics were discussed, thus leading to HCP’s preferences for telephone rather than video services [24].

#### Support for the Family

Parents valued how eHealth systems increased their control over their homecare situations [25], which was also acknowledged by HCP [24]. Parents reported increased control compared with the usual care, wherein they felt that HCP possessed greater control. eHealth also allows families to share information about their dynamics, and 1 study found an increase in family empowerment among 19 families [25]. Although primary health care services are often responsible for homecare, the families valued their contact with their health care specialist —a contact that was enabled and enhanced through eHealth. These contacts were based on the needs of the families regardless of their physical distance from the hospitals. HCP in specialist care reported that they valued the ability to be *invited* into families’ homes and regarded this invitation as a privilege [24].

eHealth was considered as a possible intrusion for both sick children and their families [24]. Some HCP referred to this technology as an *unwanted guest* [24,27] that acted as a constant reminder of the sick children [27]. Furthermore, merely setting up and managing the device may represent a burden as the functionality of the technological systems relied on often costly internet access. More parents scored their finances as more negative than positive [21], and although many families already owned the necessary hardware, HCP raised concerns regarding the economic costs related to the equipment needed for home-based support [23,24]. A lack of equipment or money to purchase such equipment would hinder equal access to services for all families. HCP stressed that parents worried enough about their sick children and that costs related to technology were unwanted [24].

#### Technological Features

This theme addresses the features of eHealth, which are summarized into the following 3 categories: usability, means to communicate, and technology as a care facilitator.

#### Usability

Utilization depends on technology that has been proven beneficial for both families and HCP. Studies report that families used eHealth systems more frequently after becoming familiar with the technology. Examples were given that demonstrated how eHealth technology allowed HCP to observe breathing patterns in real life and subsequently tailor their care plans accordingly [24,25]. However, usability depended on availability [24-27]. If the system was available not through a mobile device or a laptop at home but rather a stationary computer, it needed to be placed near each child to facilitate observations and interactions. Graphical visualizations were highly valued and were used by families that valued the opportunity to register relevant data [25]. HCP suggested that they were more prepared for video consultations than phone calls as the families could observe HCP and their actions through those meetings [24].

On the technical side, barriers for use were mainly related to whether or not the users could rely on technical solutions and internet access. Latency and interrupted video transfer disturbed the consultations [26], and rigid firewalls decreased the usability of video consultations [27].

#### Means to Communicate

Technology provides families with the unique possibility to communicate with their distant health care facilities, with both real-time audibility and visibility. The families’ ability to steer their engagement with HCP was perceived as a positive contribution [24]. Communication could be enhanced, and when relevant HCP were present, all could simultaneously partake in the discussion and thus be updated regarding care plans [24]. Through video, the HCP were able to assess the families’ reactions to the suggestions they made, thus facilitating individualized care [24,27]. In more critical cases, HCP could identify the need for action through a video consultation by obtaining a clear picture of each child, and that clearly depicted how worried his or her parents were.

The optimal length of the eHealth consultations was not defined in any study. There were uncertainties regarding the appropriate balance between clinical discussions and social interactions with both patients and their families [24]. More discrepancies additionally appeared between the issues discussed by patients and families through the eHealth systems compared with face-to face consultations. Those communicating by eHealth technology more frequently discussed pain, constipation, and anorexia, whereas life-sustaining measures were discussed face to face [22]. Similarly, Harris et al [25] found that seizures, constipation, pain, and sleep problems were addressed through their eHealth program. Sensitive topics were highlighted as particular challenges for HCP in eHealth consultations who addressed potential emotional distress and experiences of being unable to comfort the patient or caregiver [24].

#### Technology as a Care Facilitator

The ability to ensure the individual needs of each child and his or her family favored eHealth [24]; likewise, HCP felt that they had greater insight into the families’ lives as they were *in their homes*. Parents acknowledged the value of the eHealth systems when identifying their children’s care needs and tracking any changes [25]. Compared with telephonic communication, eHealth systems using video consultations were emphasized as a better measure for maintaining relationships between families and HCP [24,25]. eHealth was considered a service between phone calls and face-to-face visits as well as a valuable means for coordinating care plans as several professionals can be present for and updated on a child’s status and needs [24].

#### System for Electronic Health

This theme was characterized by the structural factors needed for users to benefit from eHealth-supported homecare. We constructed the following 2 categories to explore this theme: system resources and HCP as part of the system.

#### System Resources

HCP reported that secure access and facilities were necessary premises for the safe use of eHealth technology. Safeguarding the patient’s and family’s privacy was emphasized [24,27], which concerns the technological ability to monitor families and any potential threats to their privacy. These threats include the possibility of others listening in on the consultations both inside the health care facilities and at home. Health care facilities should be soundproof, and the risk of disclosing private information during video consultations was addressed as a major barrier toward eHealth [24].

Technical assistance and guidance were needed to be available for all users, and sufficient training before the system’s start-up was emphasized [23,25,26]. Internet speed needed to be quick and uninterrupted to avoid unwanted disturbances during consultations [25]. None of the included papers discussed the integration of eHealth components with the ehealth record system used by health care services.

When utilized as intended and when all technicalities functioned properly, the eHealth system was viewed as a favorable method for consulting with families, both from the HCP’s [24,27] and families’ perspectives [25]. eHealth systems were found to be a more economical alternative for families living far from their health care facilities [23]. Air travel is costly, and compared with outpatient clinics with and without air travel, video consultations were more economic, thus demanding less time from all involved parties. Using commercialized platforms, such as the Apple iPad, was more affordable than using noncommercialized platforms [26], particularly when families already possessed the equipment required [23,26].

#### Health Care Personnel as Part of the System

Whether or not the HCP possessed a culture that accommodated change and positive perceptions of technology seemed crucial for them to benefit from eHealth [24,27]. HCP were important advocates for their peers, especially when usual care was deemed favorable. Among HCP, face-to-face consultations were generally preferred over video consultations mainly because of privacy concerns and personal preferences. Their preferences were expected to change if routines for use were sufficiently implemented and if a coordinator had the resources necessary to schedule video consultations [24,27]. In some situations, HCP found it easier to pick up the phone, which was suggested as being related to HCP being unfamiliar with the new eHealth technology [24].

eHealth was viewed as potential support for HCP, colleagues in primary health care [24], and students [27]. The increased use of eHealth technology in primary care leads to a decreased dependency on health care specialists [24]. Furthermore, HCP from primary health care may be present in the patients’ homes during the video consultations alongside specialized care personnel for guidance and peer-to-peer support, whereas students may be present at either location. However, concerns were raised regarding how many professionals should be involved in a consultation, risking to involve more HCP than needed [24].

## Discussion

### Principal Findings

This systematic mixed methods review summarizes the research in eHealth for communication and support in home-based pediatric palliative care. We identified merely 7 eligible studies and developed 3 themes to describe our findings according to our aim, including *eHealth at home*, *technological features*, and *system for eHealth*. Generally, HCP’s voices were stronger than those of the patients and their families. eHealth systems were perceived as both a support and a possible intrusion into the home for patients and their families. Furthermore, eHealth systems needed to be easy to use and effective to facilitate communication and support.

Despite our inclusion criteria being open for all designs, we nevertheless did not identify a larger number of eligible studies. The lack of research is among the main findings of this systematic review; 4 papers included in our review were based on the same research project and sample [21-24] and were performed by the same research group who also performed the only existing systematic review on eHealth and home-based pediatric palliative care conducted before ours [6]. This review identified merely 6 eligible pediatric studies. A Cochrane review on eHealth interventions to support mental health in children with long-term physical diseases also identified a limited number of studies of low methodological quality [34], which underlines our finding that research in this area, particularly intervention studies, is scarce. Several explanations can be offered for this lack of research. Previous discussions suggest that research in this vulnerable patient group is challenging to conduct [6]—a finding that was emphasized in 2 studies included in our review [21,25].

Studies in palliative care—particularly randomized controlled trials (RCTs)—place ethical demands on research design. In controlled trials, it is important to ensure that patients receive the best care regardless of the group to which they are randomized. Randomizing patients to a waiting list is one alternative design strategy that provides all participants with the possibility to receive intervention. However, each patient’s life expectancy is limited, and any delay may equate with a patient not receiving an intervention at all. Participant recruitment and attrition are among the major barriers in pediatric palliative care studies [13], which became apparent in 1 study that was required to prematurely abandon its recruitment because of patient deaths [21]. Moreover, attrition poses obvious consequences for follow-up. Timing the measurement is challenging because of the unpredictable development of an illness. A pragmatic solution to the ethical and practical issues related to recruiting and assessing children in palliative care and their families involves recruiting HCP instead. Letting HCP reflect on the efficacy and usefulness of eHealth provides the research field with at least some information; however, eHealth consultations narrated by HCP do not represent the subjective views of children and their parents. The 2 qualitative studies [24,27] that provided this review with the richest data exclusively included HCP. Consequently, the results are largely based on expressions from HCP that represent their experiences and interpretations of the families’ views. As a result, the unique and lived experiences of each child and his or her family are lacking, thus representing a major gap in the research field.

Conducting rigorous eHealth intervention studies has also been demonstrated as methodologically challenging [35-37]. Software is intended to change and progress, which is not compatible with long-lasting, standardized, randomized trials. It has been argued that eHealth interventions are complex interventions that benefit from alternative evaluation designs [35]; nevertheless, most studies in this field are RCTs. In their previous review, Bradford et al [6] highlighted the need for alternative designs in pediatric palliative care evaluation, and our findings confirm that this need persists. Previous research has called for a pediatric palliative care study framework to establish methods to increase recruitment and decrease attrition, while simultaneously maintaining ethical issues [13]—a demand that remains highly relevant. The lack of a methodic consensus on the evaluation methods in eHealth studies adds to the difficulties associated with conducting pediatric palliative care–centered research.

Although the results of this review are characterized by few and diverse eligible studies, the findings complement existing knowledge that has been summarized by Bradford et al’s review (conducted before ours) [6]. Previous research suggests that eHealth must be a feasible means to provide information, education, and support [38,39], but the barriers associated with establishing a holistic and integrated, permanent eHealth system service seem to remain. Our review suggests that the reluctance toward eHealth technology mainly originates from HCP and to a lesser extent reflects the barriers described by the affected children and their families. The disadvantages of eHealth are related to its technological features, although the organizational structure of the health care system within which eHealth technology is placed can largely reduce these disadvantages. For eHealth technology to be integrated into standard care, health care services and HCP must acknowledge the system. HCP’s knowledge and perceptions as well as the culture within health care services will, namely, pose consequences for the adoption and utilization of eHealth services. If HCP are reluctant and prefer telephone calls for remote consultations, the integration of eHealth technology for communication and support in home-based pediatric palliative care will not be facilitated. The perceptions of children and families are additionally crucial, and positive experiences with eHealth can facilitate the use of eHealth in home-based pediatric palliative care.

Regulations meant to safeguard patients can end up withholding viable implementation of technology, and current regulations must be updated to meet the needs of a new generation of technology and users [40]. Concerns related to security and privacy in eHealth technology might be a barrier toward its development and implementation; interestingly, the integration of eHealth systems with ehealth records is not discussed in some studies, although previous research has highlighted these challenges [39,41,42].

New technology produces limitless possibilities, but unless this development is guided by patients’ needs, such technology is less likely to end up as viable and feasible for patients and HCP. Thus, the assessment of users’ needs and process evaluations are crucial in the development and evaluation of eHealth systems for communication and support in pediatric palliative care. It is ethically difficult to justify evidence that informs new services wherein the significant part is not included. This review determined that eHealth was evaluated by HCP, or objectively through medical chart notes after consultations, or (less so) through children’s and families’ self-reports. HCP and parents tend to underreport the frequency and severity of symptoms compared with self-report by the affected children [43]. Although HCP mainly focus on physical symptoms, children, siblings, and parents often suffer from psychological symptoms that are not always acknowledged by HCP [44]. The lack of research on users’ needs is alarming as every child and family is unique and the subjective experiences of both are crucial for individualizing care and optimizing their quality of life.

Although we determined that children’s voices were absent, this review indicates that eHealth technology may potentially support communication between these children and HCP without their parents’ presence and subsequently facilitate the child’s autonomy. This detail is particularly important as previous research suggests that a child wants to be actively included in both his or her own care [45,46] and any decisions related to his or her health and care [47-49].

The participants’ demographic characteristics suggest that the gender perspective should be addressed in pediatric palliative care as most children and their parents were females; boys and fathers were largely absent. Merely 2 industrialized countries were represented, and the premises for eHealth in home-based pediatric palliative care might differ between industrialized and developing countries, most often as pediatric palliative care is frequently lacking in the latter [50]. However, differences may also exist within a health care system. A lack of stable internet access, necessary equipment, and digital capabilities among users may create diverse conditions for eHealth interventions. These factors may increase health inequalities.

### Limitations

Conducting systematic reviews to synthesize evidence from qualitative and quantitative methods represents an emerging field of research. Several approaches exist [14,18]; however, agreement as to which method is more appropriate is lacking. In this review, we applied rigorous search strategies, appraised eligible studies according to checklists, and analyzed our findings using well-established methods. Thus, we are confident that we have identified and synthesized existing and relevant evidence, although other method choices may have possibly provided us with alternative results.

The wide use of eHealth and health technology terms posed consequences for our systematic search. *Internet*, *Web-based*, and *app* are terms we experienced as challenging to handle during the search process. In this context, the abbreviation *PC* can represent both personal computer and palliative care, and the result was such that, in search strings, we would miss out the combination of the two. Owing to few relevant search results and the risk of excluding relevant studies, we decided to search with broader terms in addition to these narrower terms.

### Conclusions

The scarce amount of research in the area involving eHealth-supported, home-based pediatric palliative care and the methodological and ethical challenges involved affected the conclusions that could be drawn from this mixed methods review. The results in the primary studies were mainly based on information from HCP. For eHealth to complement pediatric palliative care at home, we need research that identifies the needs and wishes of both children and their families. eHealth poses many possible advantages and can play an important role in home-based pediatric palliative care. If measures are not taken to establish a consensus on satisfactory research methods, then eHealth technology may be implemented without undergoing proper evaluation.

The findings of this review can specially inform future research through the need for a prioritization of research within eHealth to support home-based pediatric palliative care, because of the limited knowledge regarding the affected children and their families’ needs and wishes concerning eHealth. There is a need to develop research strategies to reduce unnecessary burdens on the children and their caregivers and simultaneously strive to optimize the study design.

## Appendix

Multimedia Appendix 1 Medical Literature Analysis and Retrieval System Online search string.

Multimedia Appendix 2 Methodological appraisal assessed with checklists from Joanna Briggs Institute.

## Abbreviations

eHealth: electronic health
HCP: health care personnel
RCT: randomized controlled trial
SPIDER: sample, phenomenon of interest, design, evaluation, and research type
