# Supplementary material for: Home-Based Pediatric Palliative Care and Electronic Health: Systematic Mixed Methods Review
Source: J Med Internet Res. 2020 Feb 28;22(2):e16248. doi: 10.2196/16248 (PMC7070344; doi:10.2196/16248)
Supplement: Multimedia Appendix 1 [file jmir_v22i2e16248_app1.docx]

Appendix Medline Search string

| # | Searches | Results |
| --- | --- | --- |
| 1 | Telemedicine/ | 17966 |
| 2 | Telepathology/ | 830 |
| 3 | Telerehabilitation/ | 136 |
| 4 | Remote Consultation/ | 4788 |
| 5 | Videoconferencing/ | 1285 |
| 6 | Webcasts as Topic/ | 296 |
| 7 | Medical Informatics/ | 11068 |
| 8 | Telecommunications/ | 4828 |
| 9 | Wireless Technology/ | 2928 |
| 10 | Mobile Applications/ | 2739 |
| 11 | Cell Phones/ | 7547 |
| 12 | Smartphone/ | 1632 |
| 13 | Internet/ | 66830 |
| 14 | computer communication network/ | 13917 |
| 15 | computers/ | 53605 |
| 16 | microcomputers/ | 15117 |
| 17 | computers, handheld/ | 3297 |
| 18 | (telemedicine or tele medicine or telehealth or tele health or telecare or tele care or telecommunicat* or tele communicat* or teleconferenc* or tele conferenc* or teleconsultat* or tele consultat* or telenursing or tele nursing or telemonitoring or tele monitoring or teletherap* or tele therap* or telecardiolog* or tele cardiolog* or teledermatolog* or tele dermatolog* or telediagnos* or tele diagnos* or telepatholog* or tele patholog* or telepsychiatr* or tele psychiatr* or telepsycholog* or tele psycholog* or telerehab* or tele rehab*).tw,kw,kf. | 22268 |
| 19 | (ehealth or e health or emedicine or e medicine or erehabilitation* or e rehabilitation* or mhealth or m health or ((electronic or mobile or digital) adj health)).tw,kw,kf. | 19220 |
| 20 | (((information or communicat*) adj technolog*) or hit).tw,kw,kf. | 37181 |
| 21 | application of technolog*.tw,kw,kf. | 423 |
| 22 | ((mobile or telephone or remote* or virtual* or wireless) adj3 (rehabilitat* or consultat* or counselling or communicat* or team*)).tw,kw,kf. | 6322 |
| 23 | ((mobile or portable or wireless or wearable) adj3 (device* or technolog*)).tw,kw,kf. | 11673 |
| 24 | ((mobile or portable) adj3 application*).tw,kw,kf. | 2843 |
| 25 | (smartphone* or smart phone* or mobilephone* or mobile phone* or cellphone* or cell* phone* or personal digital assistant or palm pilot*).tw,kw,kf. | 15343 |
| 26 | (webbased or web based or app or apps or ipad* or skype or internet or laptop* or web camera* or videoconferenc* or video conferenc* or touchscreen* or touch screen* or computer* or microcomputer* or pc or pcs or tablet*).tw,kw,kf. | 495558 |
| 27 | ((medical or health or nursing) adj Informatics).tw,kw,kf. | 5057 |
| 28 | ((medical adj (computer or information) adj science*) or health care technolog* or health technolog*).tw,kw,kf. | 6583 |
| 29 | (connected car* or ehomecare or e homecare or e home care or telehomecare or tele homecare or tele home care or smarthome technolog* or smart home technolog*).tw,kw,kf. | 246 |
| 30 | or/1-29 | 672847 |
| 31 | palliative care/ | 52692 |
| 32 | advance care planning/ | 2051 |
| 33 | terminal care/ | 26949 |
| 34 | terminally ill/ | 6563 |
| 35 | palliat*.tw,kw,kf. | 71455 |
| 36 | (advance* adj3 planning).tw,kw,kf. | 3212 |
| 37 | (advance* adj (disease* or illness*)).tw,kw,kf. | 19672 |
| 38 | (terminal adj (care or period)).tw,kw,kf. | 2092 |
| 39 | terminal* ill*.tw,kw,kf. | 7274 |
| 40 | ((life limit* or life threatening) adj3 (disease* or condition* or illness*)).tw,kw,kf. | 16709 |
| 41 | (end of life or dying).tw,kw,kf. | 49314 |
| 42 | or/31-41 | 179346 |
| 43 | 30 and 42 | 4258 |
| 44 | adolescent/ or child/ or child, preschool/ or infant/ or infant, newborn/ or infant, low birth weight/ or infant, small for gestational age/ or infant, very low birth weight/ or infant, extremely low birth weight/ or infant, postmature/ or infant, premature/ or infant, extremely premature/ or Disabled Children/ or Pediatrics/ or young adult/ | 3817407 |
| 45 | (child* or Infan* or minor or minors or pediatric* or paediatric* or newborn* or new born* or baby or babies or neonat* or kid or kids or toddler* or adoles* or preadoles* or teen* or boy* or girl* or underage* or under age* or juvenil* or youth* or puber* or prepuber* or pubescen* or prepubescen* or schoolchild* or preschool* or young adult* or young people).tw,kw,kf. | 2615738 |
| 46 | or/44-45 | 4689829 |
| 47 | 43 and 46 | 676 |
| 48 | limit 47 to yr="2012 -Current" | 334 |
