# Supplementary material for: Home-Based Pediatric Palliative Care and Electronic Health: Systematic Mixed Methods Review
Source: J Med Internet Res. 2020 Feb 28;22(2):e16248. doi: 10.2196/16248 (PMC7070344; doi:10.2196/16248)
Supplement: Multimedia Appendix 2 [file jmir_v22i2e16248_app2.docx]

**Appendix table 2. Methodological appraisal assessed with checklists from Joanna Briggs Institute**

| **Checklist for Quasi-experimental studies** | Bradford et al. 2012 | Harris et al. 2016 | Katalinic et al. 2013 |
| --- | --- | --- | --- |
| Is it clear in the study what is the ‘cause’ and what is the ‘effect’ | Yes | Yes | Yes |
| Were the participants included in any comparisons similar? | Unclear | NA | NA |
| Were the participants included in any comparisons receiving similar care, other than the intervention of interest? | Yes | NA | NA |
| Was there a control group? | Yes | No | No |
| Were there multiple measurements of the outcome pre and post the intervention? | No | Yes | No |
| Was follow up complete and if not, were differences between groups in terms of their follow up adequately described and analyzed? | No | NA | Unclear |
| Were the outcomes of participants included in any comparisons measured in the same way? | Yes | NA | NA |
| Were outcomes measured in a reliable way? | Yes | Yes | Unclear |
| Was appropriate statistical analysis used? | Yes | Unclear | Unclear |
| **Checklist for qualitative research** | Bradford et al. 2014 | Levy 2016 | |
| Is there congruity between the stated philosophical perspective and the research methodology? | Unclear | Unclear | |
| Is there congruity between the research methodology and the research question or objectives? | Yes | Unclear | |
| Is there congruity between the research methodology and the methods used to collect data? | Yes | Yes | |
| Is there congruity between the research methodology and the representation and analysis of data? | Yes | Unclear | |
| Is there congruity between the research methodology and the interpretation of results? | Yes | Unclear | |
| Is there a statement locating the researcher culturally or theoretically? | No | Yes | |
| Is the influence of the researcher on the research, and vice- versa, addressed? | No | No | |
| Are participants, and their voices, adequately represented? | Yes | Yes | |
| Is the research ethical according to current criteria or, for recent studies, and is there evidence of ethical approval by an appropriate body? | Yes | Unclear | |
| Do the conclusions drawn in the research report flow from the analysis, or interpretation, of the data? | Yes | Unclear | |
| Checklist for economic evaluation | Bradford et al. 2014 | | |
| Is there a well-defined question? | Yes | | |
| Is there comprehensive description of alternatives? | Yes | | |
| Are all important and relevant costs and outcomes for each alternative identified? | Yes | | |
| Has clinical effectiveness been established? | No | | |
| Are costs and outcomes measured accurately? | Yes | | |
| Are costs and outcomes valued credibly? | Yes | | |
| Are costs and outcomes adjusted for differential timing? | Yes | | |
| Is there an incremental analysis of costs and consequences? | Yes | | |
| Were sensitivity analyses conducted to investigate uncertainty in estimates of cost or consequences? | Yes | | |
| Do study results include all issues of concern to users? | Yes | | |
| Are the results generalizable to the setting of interest in the review? | Yes | | |
| Checklist for case-control studies | Bradford et al. 2014 | | |
| Were the groups comparable other than the intervention? | Yes | | |
| Were cases and controls matched appropriately? | No | | |
| Were the same criteria used for identification of cases and controls? | Yes | | |
| Was exposure measured in a standard, valid and reliable way? | Yes | | |
| Was exposure measured in the same way for cases and controls? | Yes | | |
| Were confounding factors identified? | Yes | | |
| Were strategies to deal with confounding factors stated? | No | | |
| Were outcomes assessed in a standard, valid and reliable way for cases and controls? | Unclear | | |
| Was the exposure period of interest long enough to be meaningful? | Unclear | | |
| Was appropriate statistical analysis used? | Yes | | |
